# Impact of overexpression of cytosolic isoform of *O*-acetylserine sulfhydrylase on soybean nodulation and nodule metabolome

**DOI:** 10.1038/s41598-018-20919-8

**Published:** 2018-02-05

**Authors:** Hari B. Krishnan, Bo Song, Nathan W. Oehrle, Jeffrey C. Cameron, Joseph M. Jez

**Affiliations:** 1 USDA-ARS, Plant Genetics Research Unit, 105 Curtis Hall, University of Missouri, Columbia, MO 65211 USA; 2 Division of Plant Sciences, University of Missouri, Columbia, MO 65211 USA; 3 Key Laboratory of Soybean Biology at the Chinese Ministry of Education, Northeast Agricultural University, Harbin, 150030 China; 4 Department of Chemistry and Biochemistry, University of Colorado, Boulder, CO 80309-0596 USA; 5 Department of Biology, Washington University in St. Louis, St. Louis, Missouri 63130 USA

## Abstract

Nitrogen-fixing nodules, which are also major sites of sulfur assimilation, contribute significantly to the sulfur needs of whole soybean plants. Nodules are the predominant sites for cysteine accumulation and the activity of O-acetylserine(thiol)lyase (OASS) is central to the sulfur assimilation process in plants. Here, we examined the impact of overexpressing OASS on soybean nodulation and nodule metabolome. Overexpression of OASS did not affect the nodule number, but negatively impacted plant growth. HPLC measurement of antioxidant metabolites demonstrated that levels of cysteine, glutathione, and homoglutathione nearly doubled in OASS overexpressing nodules when compared to control nodules. Metabolite profiling by LC-MS and GC-MS demonstrated that several metabolites related to serine, aspartate, glutamate, and branched-chain amino acid pathways were significantly elevated in OASS overexpressing nodules. Striking differences were also observed in the flavonoid levels between the OASS overexpressing and control soybean nodules. Our results suggest that OASS overexpressing plants compensate for the increase in carbon requirement for sulfur assimilation by reducing the biosynthesis of some amino acids, and by replenishing the TCA cycle through fatty acid hydrolysis. These data may indicate that in OASS overexpressing soybean nodules there is a moderate decease in the supply of energy metabolites to the nodule, which is then compensated by the degradation of cellular components to meet the needs of the nodule energy metabolism.

## Introduction

Soybean (*Glycine max* L. Merr) seeds contain approximately 40% protein and 20% oil and are an important source of high quality protein for both human diet and animal feed. This economically valuable legume has the ability to interact with soil-dwelling rhizobia that in compatible situations leads to the formation of unique plant organs called nodules on the roots. Nodules can be considered as ‘fertilizer units’ since they provide an ideal habitat to convert the atmospheric nitrogen into ammonia^1,2^. In the case of soybeans, ammonia is converted to ureides (allantoin and allantoic acid) and translocated to the shoots as the major nitrogen source^3^. This process of biological nitrogen fixation plays a critical role in providing nitrogen source for the synthesis of seed storage proteins^4^.

The effect of phosphorus (P) and potassium (K) on legume-rhizobia symbiosis has been previously investigated^5,6^. It is now known that both P and K have significant effect on the number of nodules as well as on plant growth^7^. Only limited information is available on the effect of sulfur (S) on legume-rhizobia symbiosis. S-deficiency results in lower nitrogen fixation^8,9^ and reduces plant growth^10^. The lower nitrogen fixation rate observed in S-deficient plants may result due to a limitation of the energy supply and a decrease in leghemoglobulin and ferredoxin concentrations^9,11,12^. This is to be expected as S is an essential element and is metabolized into the sulfur-containing amino acids and into molecules that protect plants against oxidative and environmental stresses^13,14^.

**Figure 1:**
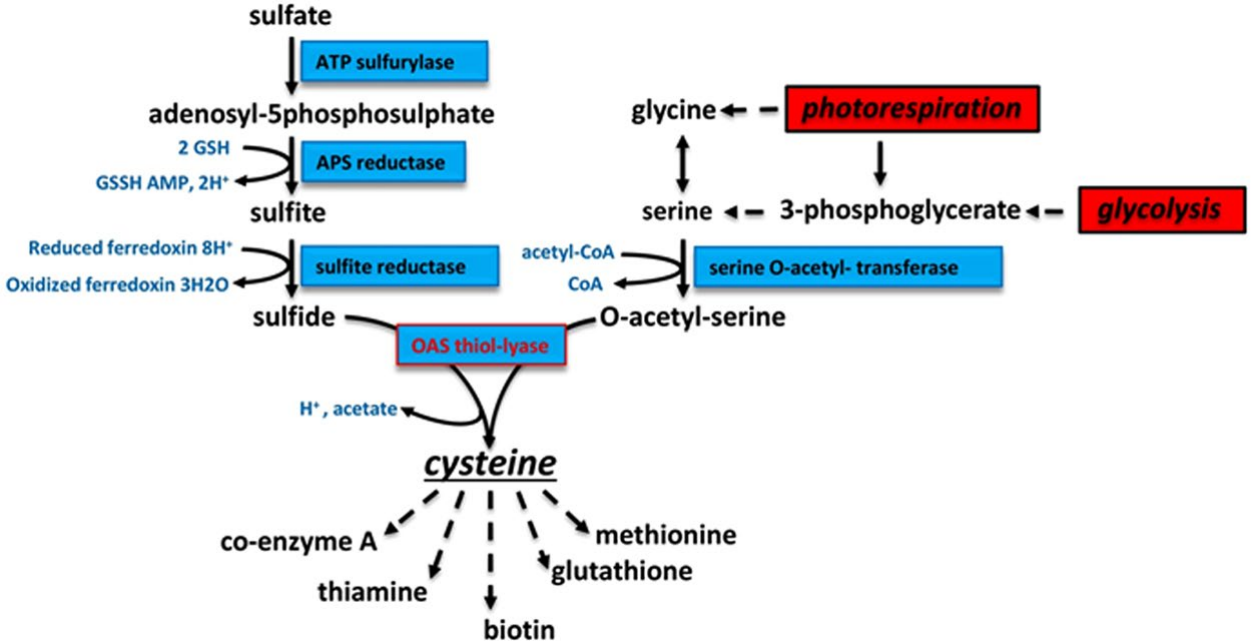
Overview of sulfur assimilation and related biochemical pathways. The sulfur assimilation pathway is shown on the left with pathways leading to O-acetylserine formation shown on the right. OASS (also, known as O-acetylserine(thiol)lyase) catalyzes the formation of cysteine from sulfide and O-acetylserine. Cysteine provides a key sulfur donor for the production of other compounds such as methionine, glutathione, and various cofactors.

Plants assimilate S from the environment and through a series of enzymatic conversions and incorporate it into metabolically useful forms (Fig. 1). Plants obtain S from the soil mainly as sulfate, a process mediated by sulfate transporters. Subsequently, through a series of enzymatic reactions the sulfate is reduced to sulfide^3^. ATP sulfurylase catalyzes the first step in the sulfur assimilation pathway leading to the activation of sulfate to 5′-adenylylsulfate (APS) (Fig. 1). In the next step, APS is converted to sulfite through the action of APS reductase. Sulfite reductase catalyzes the final reaction step in the assimilation of sulfur by converting sulfite to sulfide. Cysteine biosynthesis involves two steps: the acetylation of serine by acetyl-CoA to generate *O*-acetylserine (OAS), a reaction catalyzed by serine acetyltransferase (SAT), and the second step involves the β-replacement of the acetyl group in OAS with sulfide resulting in cysteine catalyzed by OASS. In soybean, many of the enzymes involved in these processes have been identified and biochemically characterized^15–23^. Cysteine provides a metabolic precursor for all cellular components containing reduced sulfur including methionine, glutathione, proteins, and numerous natural products^24–26^ (Fig. 1).

Even though soybeans are an excellent protein source for both humans and livestock, they are deficient in the sulfur containing amino acids, cysteine and methionine^27^. Attempts have been made to improve the sulfur-containing amino acids in soybean by expressing heterologous sulfur-rich seed proteins^28–30^; however, this approach has resulted in marginal increase in the sulfur amino acid content of transgenic seeds. As an alternative approach we have focused our effort on genetic manipulation of enzymes involved in sulfur assimilatory pathway^31^. By overexpressing a cytosolic isoform of OASS in transgenic soybean, we obtained a 58–74% increase in protein-bound cysteine levels when compared with non-transgenic soybeans^31^. OASS activity was significantly elevated not only in the transgenic soybean seeds, but also in different organs because the transgene was constitutively expressed with the CaMV 35 S promoter^31^. Thus, the transgenic soybeans overexpressing OASS provides an opportunity to study the effect of modified sulfur metabolism on nodule development and physiology. Nitrogen-fixing nodules are important for sulfur assimilation^32^. It was noted that nitrogen-fixing nodules exhibit both high APS reductase activity and thiol content, suggesting that they could serve as a major source of S-metabolites for other organs^32^. Because of the importance of S metabolism in regulating biological nitrogen fixation, in this study we investigate the effect of overexpression of OASS, a key sulfur assimilatory enzyme, on soybean nodulation.

## Results

### Transgenic soybeans exhibit elevated levels of OASS activity in nodules

**Figure 2:**
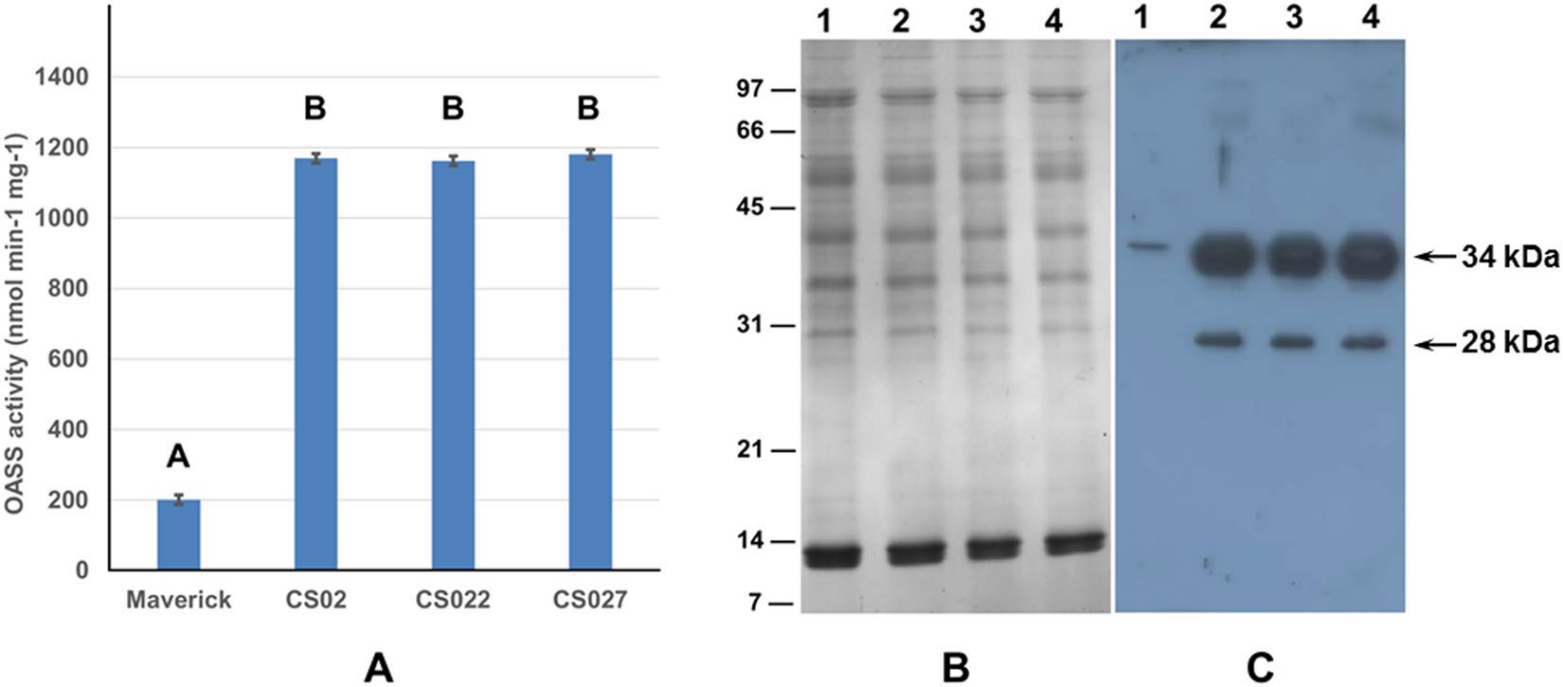
OASS activity and OASS content in soybean nodules. Protein extracts obtained from 20 day old nodules of nontransgenic and transgenic plants were assayed for OASS activity (**A**). Average values are shown ± SD (n = 3). Total nodule proteins were separated on 15% SDS-PAGE gels and either stained with Coomassie Blue (**B**) or transferred to nitrocellulose membrane and probed with antiserum against soybean OASS (**C**). Antibody reactivity was detected by using anti-rabbit IgG-horseradish peroxidase conjugate followed by chemiluminescent detection. Lane 1 nodule protein from non-transformed wild-type, lanes 2 to 4 nodule protein from OASS transformed transgenic plants CS02, CS022, and CS027, respectively. Autoradiographs were scanned with an Epson Perfection V700 PHOTO, with image acquisition done through Adobe Photoshop, and scanned at 300 dpi using 24-bit color picture setting. Images were processed and cropped with PowerPoint.

Previously, we reported OASS activity to be elevated in root, stem, inflorescence, leaves, and seeds of transgenic soybeans when compared to non-transformed soybean plants^31^. To examine if similar elevation of OASS activity also occurs in nodules, we inoculated three independent transgenic soybean plants (CS02, CS022 and CS027) with *B. japonicum* USDA110 and collected nodules at 20 days after inoculation. Measurement of OASS activity from the nodules clearly demonstrated that all three independent transgenic soybean plants overexpressing OASS had significantly higher activity than non-transformed soybean plants (Fig. 2A). OASS activity was 6-fold higher in nodules from transgenic soybean plants than the non-transformed plant (Fig. 2A). We also examined if the elevated OASS activity was due to over accumulation of OASS protein. An examination of the SDS-PAGE resolved total protein profiles of 20-day old nodules revealed no observable differences between transgenic and non-transformed control plants (Fig. 2B). Western blot analysis clearly demonstrated that transgenic soybean nodules accumulated higher amounts of OASS protein than that of non-transformed soybean nodules (Fig. 2C). In non-transformed soybean nodules the OASS antibody recognized a single 34-kDa protein while in transgenic soybean nodules two immunoreactive proteins (34 and 28 kDa) were detected (Fig. 2C). The reactivity of OASS antibody to the 28 kDa protein may be non-specific or the 28 kDa immunoreactive protein may represent a breakdown product of the mature 34 kDa OASS.

### Overexpression of OASS does not influence nodule number but negatively impacts plant growth

**Figure 3:**
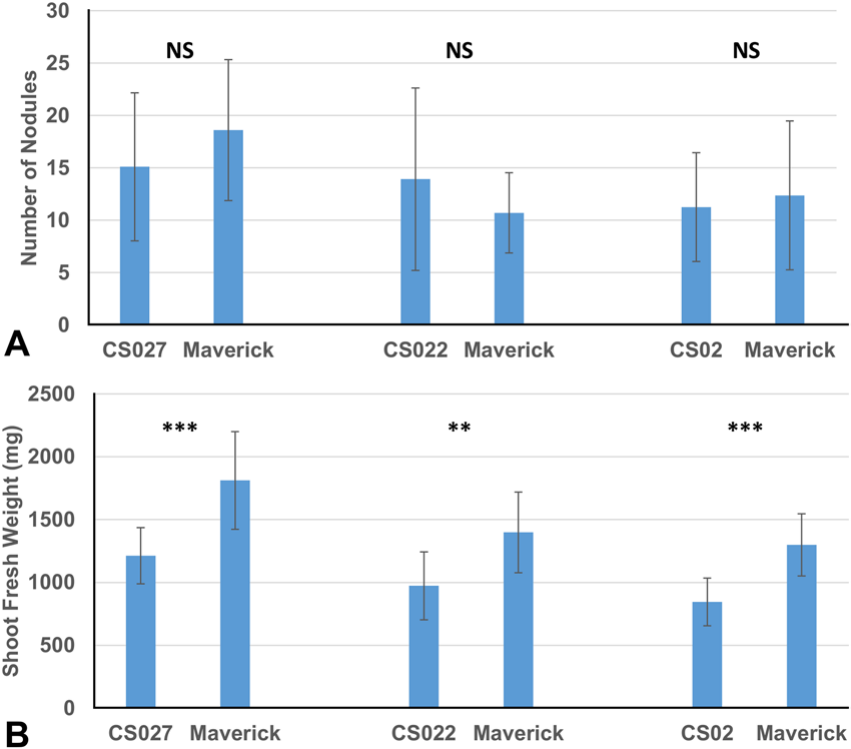
Nodulation and shoot weight of soybean plants inoculated with *Bradyrhizobium japonicum* USDA 110. Number of nodules (**A**) and shoot fresh weight (**B**). Values are presented as mean ± SE (n = 18). (P ≤ 0.05 threshold).

To ascertain if overexpression of OASS affected soybean nodulation, we inoculated 3-day-old soybean seedlings with *B. japonicum* USDA110 and counted the number of nodules formed on the roots after 20 days after inoculation. Both transgenic and non-transformed soybeans formed almost identical number of nodules (Fig. 3A). In contrast to the nodule number, examinations of the shoot fresh weight at 20 days after inoculation (Fig. 3B) revealed that transgenic soybean plants were negatively impacted. The shoot fresh weight of the OASS overexpressing transgenic soybean plants were significantly lower than the non-transformed soybean plants (Fig. 3B). One of these transgenic event (CS02) has been grown for several generations and the resulting homozygous transgenic plants have been previously characterized^31^. This transgenic event (CS02) was selected for all our subsequent studies.

### Overexpression of OASS and breakdown of nodule structural integrity

**Figure 4:**
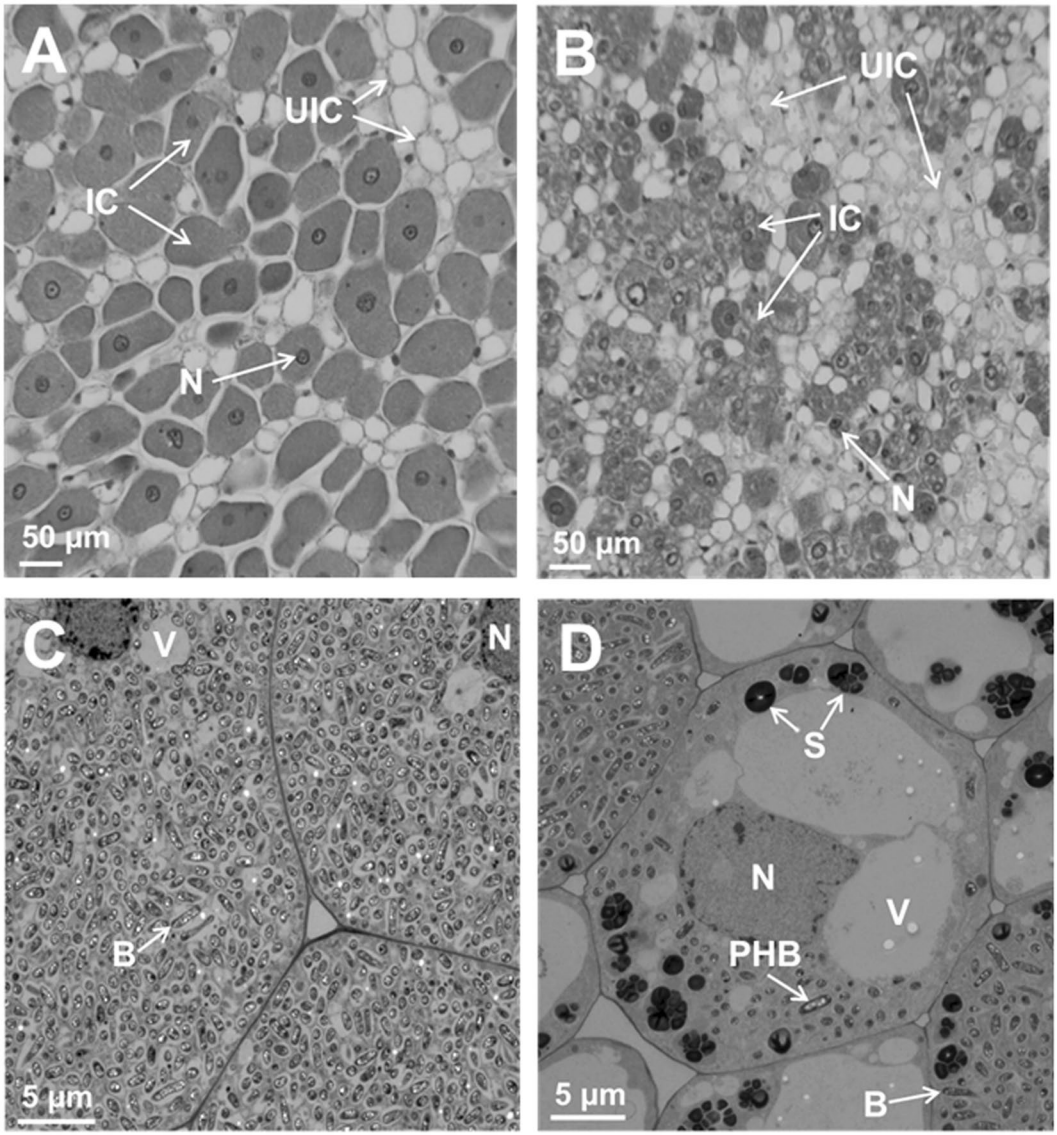
Anatomy of soybean nodules. Nodules collected at 15 days after infection were thick-sectioned and viewed under light microscope (**A**,**B**). For ultrastructral observation thin-sections were examined by electron microscopy (**C**,**D**). Note the cells from non-transformed wild-type nodules are filled with bacteria (**A**,**C**) while the cells from OASS overexpressing transgenic nodule are not completely filled by bacteria (**B**,**D**). Also note the presence of prominent starch grains in the cells of from OASS overexpressing transgenic nodule (**D**). B, bacteroid; IC, infected cells; N, nucleus; PHB, polyhydroxybutyrate; S, starch; UIC, uninfected cell; V, vacuole.

Anatomy of nodules initiated by *B. japonicum* USDA110 on the roots of OASS overexpressing transgenic soybean plants and non-transformed soybean plants were examined by light microscopy (Fig. 4A and B). An examination of 15 days after inoculation nodules revealed anatomical features typical of soybean nodules^33^. In the case of non-transformed soybean plants, the central region of the nodule was filled with bacteria and stained darker than the surrounding regions (Fig. 4A). Whereas, in the OASS overexpressing nodules only limited number of cells in the central region was occupied by rhizobia (Fig. 4B). Larger number of uninfected cells was also seen (Fig. 4B). Transmission electron microscopic examination of thin-sections of 15 days after inoculation nodules from non-transgenic plants revealed the presence of numerous bacteroids in the infected cells. These bacteroids were surrounded by peri-bacteroid membranes (symbiosomes). At this developmental stage, a majority of symbiosomes contained a single bacteroid though some also contained more than one bacteroid (Fig. 4C). Prominent polyhydroxybutyrate (PHB) inclusions were observed in these bacteroids. Some of the infected cells in OASS overexpressing transgenic plants revealed prominent vacuoles and starch grains (Fig. 4D). In fully infected cells, the bacteroids were enclosed in symbiosomes with prominent PHB inclusions (Fig. 4D).

**Figure 5:**
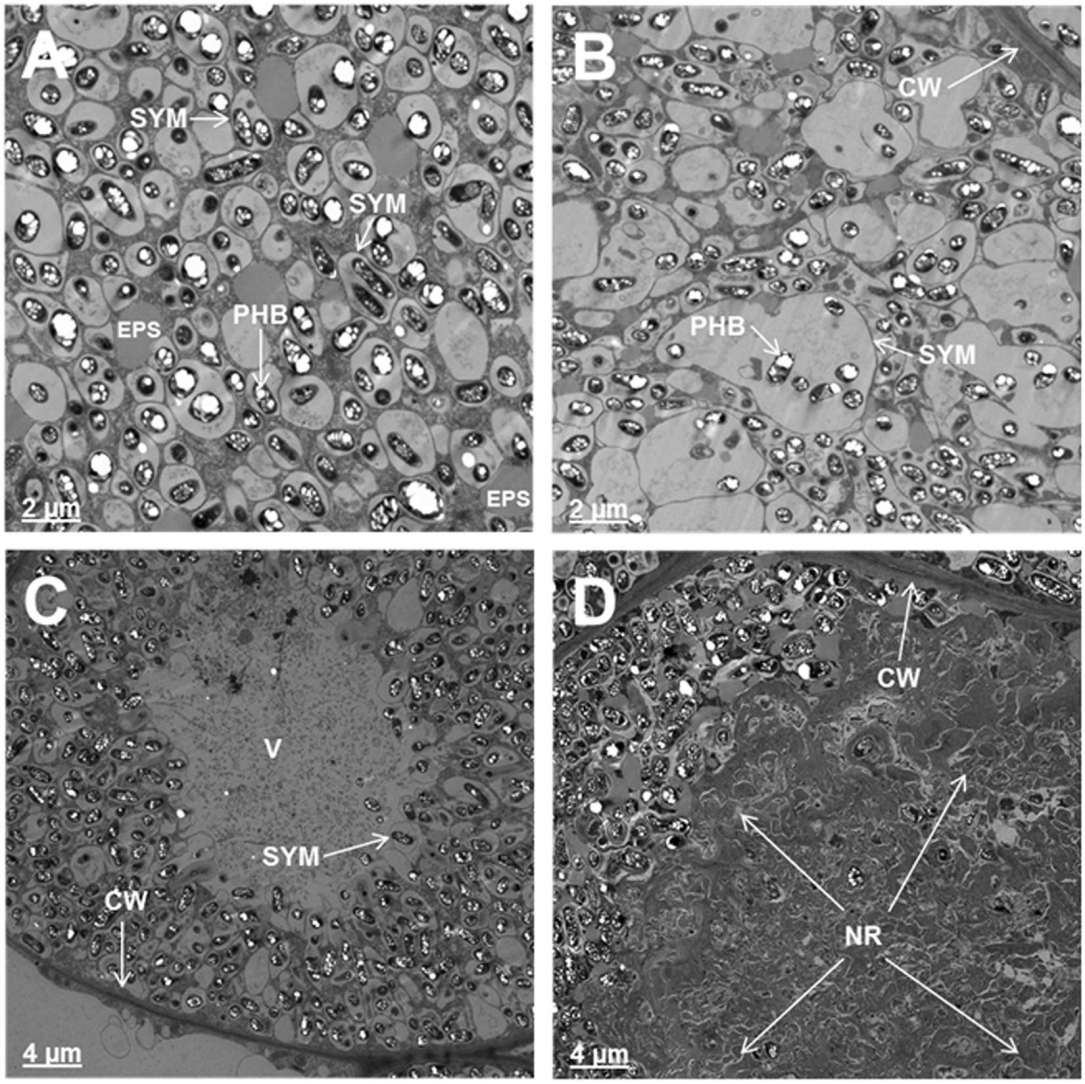
Ultrastructral alterations in 30 days after infection nodules from OASS overexpressing transgenic plants. In cells from non-transformed wild-type nodules at 30 days after infection, the symbiosomes are dilated and contain one or two bacteroids (**A**). Note the accumulation of ocular substances in several of the cells (**A**). In contrast, cells from OASS overexpressing transgenic nodules the symbiosome are enlarged into vacuolar structures containing several bacteroids (**B**). In some cases, the central region of these cells has collapsed (**C**) and accumulates granular material (**D**). CW, cell wall; EPS, extracellular polysaccharide; NR, necrotic region; PHB, polyhydroxybutyrate; Sym, symbisome; V, vacuole.

An examination of infected cells of 30 days after inoculation nodules from non-transgenic plants revealed dilated symbiosomes (Fig. 5A). Interestingly, several symbiosomes lacked bacteroids; instead were filled with inclusions, presumably extracellular polysaccharides (EPS) (Fig. 5A). The symbiosomes in the infected cells of 30 days after inoculation nodules from OASS overexpressing transgenic plants were enlarged into sack-like structures that contained several bacteroids in them (Fig. 5B). Often, these nodules also contained central vacuolated regions presumably due to the disintegration of membrane integrity (Fig. 5C). In some infected cells, the symbiosomes were completely disintegrated and the entire central region appeared as granular matrix resembling necrotic region (Fig. 5D). Such a necrotic region was not detected in 30 days after inoculation nodules from non-transgenic plants.

### Analysis of protein changes in the nodules

**Figure 6:**
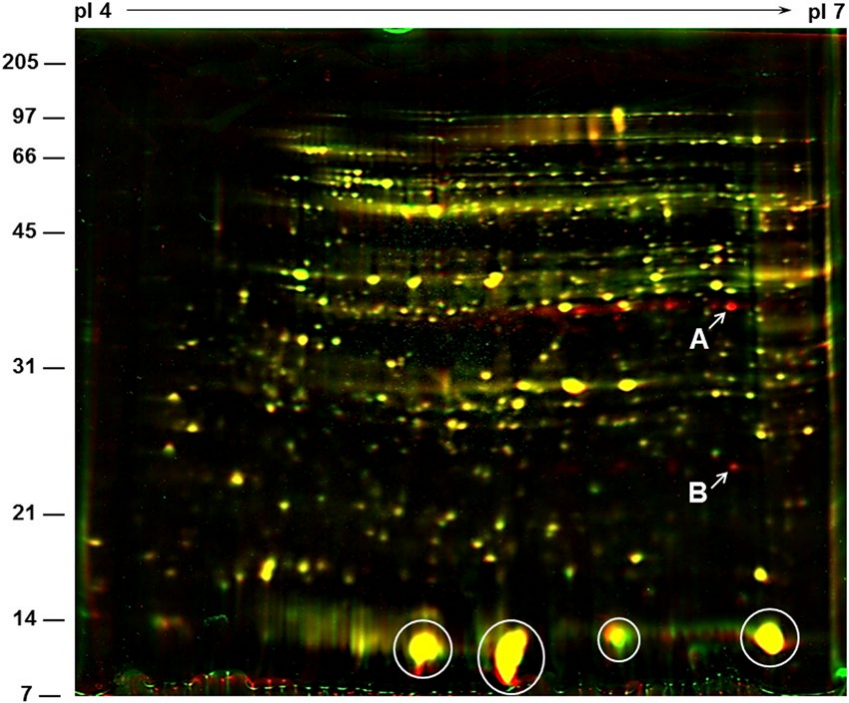
Overlay of two separate two-dimensional gels of soybean nodule proteins using Delta2D software. Nodule cytosolic proteins were separated by isoelectric focusing (pI 4–7) followed by second dimension SDS-PAGE on a 15% gel. Resolved proteins were visualized by staining the gel with colloidal Coomassie Blue G-250. Gels were scanned and resulting images were assigned two different colors (green = non-transformed wild-type nodules; red = OASS overexpressing transgenic mutant nodules) and overlaid using Delta2D software in order tvisualize the differences between the two. Yellow demonstrates similar protein quantities in each. Red color indicates a much lower abundance of that particular protein in the non-transformed wild-type nodules. MALDI-TOF-MS identified the 34 kDa protein spot (**A**) as OASS and the 22 kDa protein spot (**B**) as phosphinothricin acetyltransferase. Abundant leghemoglobulin protein spots are circled.

One-dimensional analysis of total protein from OASS overexpressing nodules revealed no observable changes when compared to non-transgenic control nodules (Fig. 2B). To facilitate the detection of any potential protein changes high-resolution 2-D gel electrophoresis was performed (Fig. 6). Soluble proteins isolated from 20 days after inoculation nodules from both the OASS overexpressing and control plants were resolved into several hundred distinct protein spots. Leghemoglobin, the most abundant protein in soybean nodule, was resolved into four prominent spots Lba, Lbc1, Lbc2, and Lbc3. Visual observation of the Coomassie G-250 stained 2-D gels indicated no major changes in the overall number of protein spots and their abundance in OASS overexpressing and control nodules. When stained gels were analyzed for proteome differences using Delta2D v3.6 image analysis software, a few unique protein spots were found in OASS overexpressing nodules (Fig. 6). Three protein spots with apparent molecular weight of 34 kDa, but with different isoelectric points (5.8, 6.2, and 6.5) were present in OASS overexpressing nodules. In addition, a 22-kDa protein spot with an isoelectric point of 6.5 was also unique to OASS overexpressing nodules. The 34 kDa (pI 6.5) and the 22 kDa (pI 6.6) protein spot were excised from the gel, digested with trypsin, and analyzed by MALDI-TOF-MS (Supplemental Table 1). Using Mascot, the empirically determined mass-to-charge ratios of the peptides were compared to known peptides in the National Center for Biotechnology Information nonreduandant database. Several peptides from the 34 kDa protein gave statistically significant protein scores for matches with soybean OASS (32% sequence coverage), with MOWSE scores above 95% confidence level (Supplemental Table 1). Similarly, four peptides from the 22 kDa protein revealed homology to phosphinothricin acetyltransferase (32% sequence coverage), which is a selection marker for transgenic plants and provide resistance to herbicide glufosinate.

### Increased cysteine, glutathione, and homoglutathione levels in nodules

**Figure 7:**
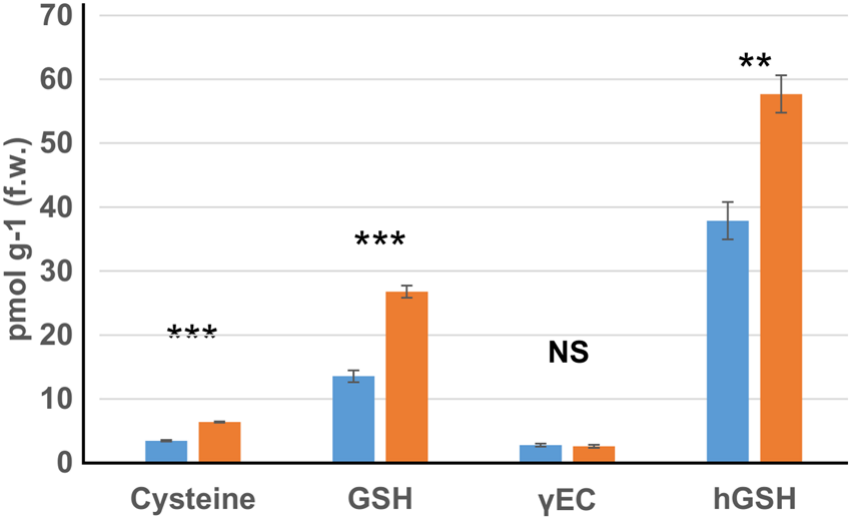
Impact of OASS overexpression on thiol metabolite levels in 30-day-old soybean nodules. Cysteine, glutathione (GSH), glutamylcysteine (γ-EC) and homoglutathione (hGSH) were determined with monobrombimane derivatization and fluorescence detection. Thiols from non-transformed wild-type and transgenic soybeans are shown by blue and orange bars, respectively. Values are presented as mean ± SE. (P ≤ 0.05 threshold).

Biological nitrogen fixation is one of the main sources for the generation of reactive oxygen/nitrogen species (ROS/RNS). To counter act against the damaging effect of the ROS/RNS, nodules utilize a rich array of antioxidant metabolites and enzymes. Prominent among the metabolites are glutathione (GSH) and its legume-specific analog homoglutathione (hGSH). It has been shown that there is an active ascorbate-GSH cycle in the root nodules, which requires a continuous supply of GSH to protect nitrogen fixation against toxic oxygen species^34–36^. We examined if there are any changes in the concentration of antioxidant metabolites due to overexpression of OASS in the nodules. Cysteine, γ-glutamylcysteine (γEC), GSH, and hGSH levels were determined by monobromobimane-derivatization and HPLC analysis. This analysis established that cysteine, GSH, and hGSH nearly doubled in OASS overexpressing nodules when compared to control non-transgenic nodules (Fig. 7), but that the concentration of γEC, a metabolic precursor to both GSH and hGSH, remained constant (Fig. 7). As shown previously^38^, hGSH was found to be the most abundant thiol metabolite in soybean nodules.

In addition to antioxidant metabolites, we also examined the levels of antioxidant enzymes in the nodules. Nitrogen-fixing nodules are important sources of ROS. In addition to the antioxidants, nodules also contain enzymes that scavenge the ROS and thereby protect the cells against oxidative damage^37^. Since transmission electron microscopy observation of 30-day-old OASS overexpressing nodules revealed breakdown of organelles and symbiosomes, we examined the levels of key antioxidant enzymes. The activity of ascorbate peroxidase (APX), catalase (CAT) and superoxide dismutase (SOD), key enzymes involved in antioxidant defense, were measured in15 and 30 days after inoculation nodules (Supplemental Table 2). No significant differences in the activity of APX and CAT were seen between OASS overexpressing and non-transgenic control nodules both in 15 and 30 days after inoculation nodules (Supplemental Table 2). In contrast, the activity of SOD was found to be elevated in OASS overexpressing nodules when compared to that of non-transgenic control nodules both in 15 and 30 days after inoculation plants (Supplemental Table 2).

### Impact of overexpression of OASS on nodule metabolites

A combination of metabolite profiling platforms was used to gain insight into the biochemical changes resulting due to overexpression of OASS in nodules. A total of 305 metabolite compounds were detected, quantified and analyzed (Supplemental dataset). This includes 72 amino acids and amino acid derivatives, 52 carbohydrates, 55 lipids, 47 peptides, 29 secondary metabolites, 23 cofactors/prosthetic groups/electron carriers, 24 nucleotides, 1 hormone, and 1 xenobiotic chemical. Out of 61 metabolites analyzed, 32 were increased and 29 decreased in OASS overexpressing nodules compared to non-transformed wild-type nodules (Supplemental Table 2). The levels of 19 metabolites were significantly different (p < 0.05) between the control and OASS overexpressing nodules with 10 higher and 9 lower in OASS overexpressing soybean nodules compared to control non-transgenic nodules (Supplemental dataset). Several other metabolites derived from serine, aspartate, glutamate, and branched-chain amino acid pathways were also significantly elevated in OASS overexpressing nodules (Table 1). The concentration of 5-oxoproline, which is involved in GSH metabolism, was 2.9-fold higher in OASS overexpressing nodules (Table 1). Modest increases in arabonate, ribitol, ribose, xylonate, and xylose were also observed in OASS overexpressing nodules. In contrast, metabolites belonging to sucrose, glucose, and fructose metabolism were noticeably lower in OASS overexpressing nodules (Table 1). A similar reduction in the levels of several flavonoids in OASS overexpressing nodules was also observed (Table 1). As expected the cysteine content of OASS overexpressing nodules was 2.2-fold higher than that of control nodules (Supplemental dataset). The concentration of serine was also slightly higher in these nodules. In contrast, the concentration of *O*-acetylserine, a key metabolite that regulates the sulfur assimilatory pathway was significantly lower in OASS overexpressing nodules (Supplemental Fig. 2).

**Table 1:** Heat map for selected metabolites with fold-changes and ANOVA significance level.

Super Pathway	Sub Pathway	Metabolite	Platform	Transgenic Nontransgenic	N Transgenic N Nontransgenic	
					p-value	q-value
Amino acid	Serine family (phosphoglycerate derived)	cysteine	GC/MS	2.19	0.0002	0.0048
		glycine	GC/MS	1.26	0.0204	0.102
		O-acetylserine	GC/MS	*0.49*	0.0022	0.0235
	Aspartate family (OAA derived)	alanine	GC/MS	**1.11**	0.075	0.1985
		beta-alanine	GC/MS	1.66	0.0012	0.0163
		methionine sulfoxide	LC/MS pos	*0.83*	0.032	0.1297
		pipecolate	GC/MS	1.49	0.0451	0.1506
	Glutamate family (alpha-ketoglutarate derived)	4-acetamidobutanoate	LC/MS pos	1.28	0.01	0.0683
		argininosuccinate	LC/MS pos	**1.23**	0.062	0.1808
		gamma-aminobutyrate (GABA)	GC/MS	1.28	0.0348	0.1325
		histidine	LC/MS neg	*0.79*	0.0026	0.0252
		N-acetylglutamate	LC/MS pos	1.45	0.0004	0.0091
		N-alpha-acetylornithine	LC/MS pos	*0.94*	0.0158	0.0899
		N-acetylproline	LC/MS pos	1.68	0.0029	0.0267
		trans-urocanate	LC/MS pos	2.44	3.07E-06	0.0006
		N-methyl-4-aminobutyric acid	LC/MS pos	***0.87***	0.0653	0.1856
		4-hydroxy-2-oxoglutaric acid	GC/MS	*0.64*	0.008	0.0572
	Branched Chain Amino Acids (OAA derived)	isoleucine	LC/MS pos	1.19	0.0423	0.1492
	Branched Chain Amino Acids (pyruvate derived)	leucine	LC/MS pos	1.14	0.0146	0.0894
		valine	LC/MS pos	1.2	0.041	0.149
	Glutathione metabolism	5-oxoproline	LC/MS neg	2.87	1.84E-05	0.0018
Carbohydrate	Glycolysis	1,3-dihydroxyacetone	GC/MS	*0.8*	0.019	0.1006
		glucose	GC/MS	*0.74*	0.0209	0.102
		lactate	GC/MS	1.23	0.0072	0.0546
	TCA cycle	alpha-ketoglutarate	GC/MS	***0.69***	0.0787	0.1999
		malate	GC/MS	*0.79*	0.0187	0.1006
	Amino sugar and nucleotide sugar	arabonate	GC/MS	1.32	0.0237	0.1086
		ribitol	GC/MS	1.25	0.0332	0.1319
		ribose	GC/MS	1.19	0.0346	0.1325
		xylonate	GC/MS	1.5	0.0006	0.0103
		xylose	GC/MS	1.32	0.0158	0.0899
	Inositol metabolism	chiro-inositol	GC/MS	***0.85***	0.0277	0.12
		myo-inositol	GC/MS	*0.85*	0.05	0.1537
	Sucrose, glucose,fructose metabolism	fructose	GC/MS	*0.59*	0.0036	0.0299
		maltose	GC/MS	*0.68*	0.0113	0.0743
		sucrose	LC/MS neg	*0.7*	0.0007	0.0103
Lipids	Free Fatty Acid	2-hydroxyglutarate	GC/MS	***0.87***	0.0917	0.2268
		8-hydroxyoctanoate	LC/MS neg	*0.81*	0.0223	0.1063
		caprylate (8:0)	LC/MS neg	*0.76*	0.039	0.1458
		dihomo-linoleate (20:2n6)	LC/MS neg	**1.25**	0.0974	0.2371
		margarate (17:0)	LC/MS neg	*0.74*	0.0466	0.1531
		oleate (18:1n9)	GC/MS	1.39	0.0446	0.1506
	Oxylipins	13S-hydroperoxy-9Z,11E,15Z-octadecatrienoate	LC/MS pos	*0.84*	0.0245	0.1086
	Glycerolipids	2-linoleoylglycerol (2-monolinolein)	LC/MS neg	**1.64**	0.0767	0.1999
		1-behenoylglycerol (1-monobehenin)	GC/MS	*0.63*	0.0435	0.1506
		1-palmitoylglycerophosphoinositol*	LC/MS neg	2.78	0.0025	0.0247
		2-oleoylglycerophosphocholine*	LC/MS pos	**1.76**	0.0782	0.1999
	Choline metabolism	choline phosphate	LC/MS pos	1.47	6.89E-05	0.0022
		ethanolamine	GC/MS	1.35	0.0197	0.1013
	Sterols	3-hydroxy-3-methylglutarate	GC/MS	*0.85*	0.0415	0.149
		beta-sitosterol	GC/MS	*0.88*	0.0821	0.2057
		campesterol	GC/MS	*0.78*	0.0291	0.1204
Cofactors, Prosthetic Groups, Electron Carriers	Nicotinate and nicotinamide metabolism	nicotianamine	LC/MS pos	*0.86*	0.0284	0.1203
		nicotinate	GC/MS	1.18	0.0494	0.1537
		riboflavin (Vitamin B2)	LC/MS pos	1.16	0.002	0.0224
		flavin mononucleotide (FMN)	LC/MS neg	*1.18*	0.0724	0.198
	Vitamin B metabolism (B6 or B12)	pyridoxate	LC/MS neg	***0.88***	0.0145	0.0894
Nucleotide	Purine metabolism	adenine	LC/MS pos	1.14	0.0081	0.0572
		allantoin	GC/MS	*1.16*	0.0738	0.198
		guanine	LC/MS pos	*0.84*	0.0484	0.1537
		urate	GC/MS	1.44	0.0495	0.1537
		xanthine	GC/MS	**1.24**	0.0585	0.1741
	Pyrimidine metabolism	cytidine	LC/MS pos	1.35	6.95E-05	0.0022
		cytosine	GC/MS	1.49	0.016	0.0899
		ectoine	LC/MS pos	***0.68***	0.0627	0.1808
		uracil	GC/MS	1.32	0.0052	0.0409
	Flavonoids	6, 7, 4′-trihydroxyisoflavone	LC/MS pos	*0.62*	0.0007	0.0103
		apigenin	LC/MS neg	***0.46***	0.0736	0.198
		daidzein	LC/MS pos	*0.83*	0.0006	0.0103
		daidzin	LC/MS pos	*0.62*	0.0014	0.0171
		genistein	LC/MS neg	*0.84*	5.52E-05	0.0022
		glycitein	LC/MS pos	*0.84*	0.0036	0.0299
		glycitin	LC/MS neg	*0.59*	3.65E-05	0.0022
		chrysoeriol	LC/MS neg	**1.07**	0.0993	0.2371
		glyceollin III	LC/MS neg	***0.48***	0.0561	0.1696
	Terpenoids	alpha-amyrin	GC/MS	***0.81***	0.0996	0.2371

Italic: indicates significant difference (*p* ≤ 0.05) between the groups shown, metabolite ratio of <1.00.

Bold/italic: narrowly missed statistical cutoff for significance 0.05 < p < 0.10, metabolite ratio of <1.00.

Underline: indicates significant difference (*p* ≤ 0.05) between the groups shown; metabolite ratio of ≥1.00.

Bold Underline: narrowly missed statistical cutoff for significance 0.05 < p < 0.10, metabolite ratio of ≥1.00.

### Overexpression of OASS alters flavonoid and isoflavonoid levels

**Figure 8:**
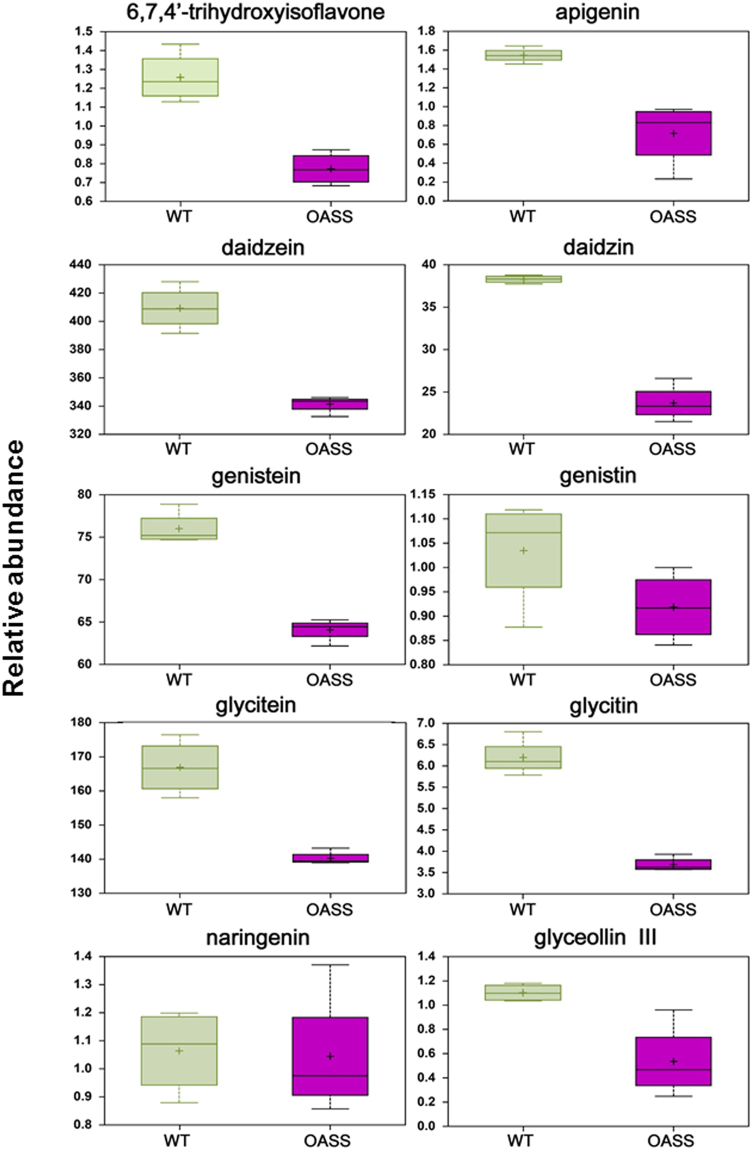
Changes in the abundance of flavonoids in non-transformed wild-type and transgenic soybean nodules. Relative abundance of metabolites is shown on y-axis. Box plots were generated for compounds with significant increase or decrease using both t-test and FDR, with p < 0.05 and q < 0.10 as significant values.

The process of nodulation involves extensive signal exchange between the legumes and rhizobia. Flavonoids released by legume roots are crucial signaling molecules in the symbiosis. In the case of soybean, isoflavones daidzein, genistein, and glycitein are the primary signal molecules that induce the transcription of *nod* genes leading to the production of Nod factors^39^. Interestingly, we found striking differences in the flavonoid and isoflavonoid levels between the OASS overexpressing and control soybean nodules (Fig. 8). Eight of ten detected flavonoids and isoflavonoids (6,7,4′-trihydroxyisoflavone, apigenin, daidzein, daidzin, genistein, glycitein, glycitin, and glyceollin III) were reduced 0.4– to 0.8 fold in nodules from plants overexpressing OASS. The levels of genistin and naringenin, which are in low abundance, were similar between OASS overexpressing and control non-transgenic soybean nodules (Fig. 8).

## Discussion

OASS, which catalyzes the formation of cysteine using sulfide and the carbon backbone provided by *O*-acetylserine, is central to the assimilation process of sulfur in plants. Recent work by Kalloniati *et al*. (2015)^32^ demonstrated that nitrogen-fixing nodules are also the predominant sites for cysteine accumulation, which leads to whole-plant reprogramming of sulfur metabolism. In that work, high thiol content observed in the nodules was linked to active biological nitrogen fixation. It was reported that non-nitrogen fixing (Fix^−^) nodules had significantly lower content of cysteine than functional (Fix+) nodules. In our study, we observed overexpressing of OASS in soybean resulted in elevated cysteine content in nodules (Fig. 7). Yet, these plants were affected in plant growth suggesting that just an overall increase in the thiol metabolite content alone may not be sufficient for improved biological nitrogen fixation and plant growth.

Light microscopy analysis reveals that OASS overexpressing nodules are defective in differentiation of infected cells. The most visible feature of infected cell differentiation is the increase in the cell volume. The growth of infected cell is accompanied by several rounds of endoreduplication. Following the maturation of cells the rhizobia differentiate into bacteroids (Fig. 4A). However, in OASS overexpressing nodules this process is delayed and most of these cells remain meristematic (Fig. 4B). Factors that inhibit endoreduplication could result in a decreased cell size and cause early senescence and disintegration of symbiosomes^40^. Transmission electron microscopy observation clearly demonstrates that OASS overexpressing nodules reveal disintegration of symbiosome membrane (Fig. 5). It was earlier reported that during nodule senescence the symbiosome membrane is first targeted for degradation^40,41^. Additionally, in senescing soybean nodules levels of hydrogen peroxide (H_2_O_2_) and lipid hydroperoxides increase, leading to generation of ROS at relatively high rates^42^. ROS-related enzymes are very active in legume nodules and play an important role in protecting the cells from oxidative damage by scavenging ROS/RNS^37,38^. Since our ultrastructural investigation revealed significant membrane disintegration, we examined if this could be due differences in the ROS-related enzyme activity in nodules. Interestingly, no significant differences in the activity of APX, CAT, and SOD were observed between nontransgenic and transgenic soybean (Supplemental Table 1). It is possible that the elevated levels of GSH and hGSH (Fig. 7) may compensate by providing additional redox buffering capacity. Overall, the membrane disintegration observed in OASS overexpressing nodules is not due a paucity of antioxidant enzyme activity or thiol metabolites needed for scavenging ROS/RNS.

Metabolic profiling revealed striking differences in several metabolites between OASS overexpressing and control non-transgenic soybean nodules (Table 1; Figs 7 and 8; Supplemental Fig. 1). In addition to the thiol-containing metabolites (Fig. 7), we examined serine and O-acetylserine in nodules (Supplemental Fig. 2). Levels of *O*-acetylserine, a substrate of OASS, were reduced in nodules, as would be expected by the over-expression of OASS and increased cysteine production. Serine, the precursor of *O*-acetylserine, and methionine, which uses cysteine in its biosynthesis, were not altered in nodules. We also found the levels of isoflavonoids were significantly lowered in OASS overexpressing nodules compared to control non-transgenic soybean nodules. Similar situation was also reported in *Fusarium virguliforme* infected roots of soybean^43^. It was proposed that degradation of these isoflavonoids may facilitate root necrosis observed in sudden death syndrome^43^. We also observed tissue necrosis in 30 day old OASS overexpressing nodules.

The carbon skeleton for sulfur assimilation is provided by *O*-acetylserine, an activated form of serine, which in turn is biosynthesized from 3-phosphoglycerate, an intermediate of glycolysis. Glycolysis and the tricarboxylic acid (TCA) cycle, provide carbon skeletons for the production of amino acids and its derivatives, in addition to their role in energy metabolism. Aromatic amino acids, branched-chain amino acids (BCAA), and alanine levels were decreased in OASS overexpressing soybean nodules than in non-transgenic control plants (Table 1; Supplemental Fig. 1). These amino acids derive their carbon backbone from phosphoenolpyruvate and pyruvate, which are downstream from 3-phosphoglycerate, from which serine is derived. It is possible that the need for additional carbon required by the higher activity of OASS causes a limitation in the availability of glycolytic metabolites for amino acid biosynthesis. Because the partitioning of glycolytic and TCA cycle intermediates is finely controlled^44^, these metabolite changes may reflect balancing of inputs between energy metabolism and carbon for nitrogen and sulfur assimilation. Any change in the requirement for carbon for one purpose, *e.g*. for increase production of serine required for the higher activity of OASS, is expected to cause a perturbation in overall carbon partitioning, as observed in our metabolite profile data (Table 1; Supplemental Fig. 2).

Several pieces of the metabolic profiling data suggest a possible lower level of energy supply to the nodule in the plants where carbon in the leaf is directed towards greater assimilation of sulfur. In nodules from OASS overexpressing soybean nodules, we observed lower levels of glucose, fructose, sucrose and maltose and a higher level of lactate, than in non-transgenic plants (Table 1; Supplemental Fig. 1). In addition, the pentose sugars ribitol, ribose, xylonate, and xylose, as well as C4 arabinates, possibly derived from cell wall turnover, were higher in OASS overexpressing soybean nodules compared to non-transgenic plants nodules. Likewise, BCAA levels were also higher in the OASS overexpressing soybean nodules compared to non-transgenic nodules. The elevated levels of BCAA were most likely due to an increase in overall protein degradation rather than amino acid biosynthesis, as we also observed an increase in 4-acetamidobutanoate, pipecolate, and trans-urocanate, which are degradation products of putrescine, lysine, and histidine, respectively. Likewise, the decreased levels of flavonoids and isoflavonoids suggest a general decrease in carbon metabolisms (Fig. 8). Increased levels of several N-acetyl amino acids observed in the OASS overexpressing soybean nodules also suggest elevated proteolysis in OASS overexpressing soybean nodules plants relative to the non-transgenic plants, as co-translational acetylation of the N-terminal amino acid is a common protein modification found in eukaryotes^45^. Taken together these data may indicate that in OASS overexpressing soybean nodules, there is a moderate decease in the supply of energy metabolites to the nodule, which is then compensated by the degradation of cellular components to meet the needs of the nodule energy metabolism. Ultimately, these changes negatively impact soybean growth. Even though constitute overexpression of OASS resulted in elevated levels of sulfur-containing amino acids in soybean seeds^31^, it accompanied by a reduction in the plant growth. To overcome this negative effect, it might be desirable to utilize seed-specific promoter to achieve the goal of improving the sulfur amino acid content of the seed.

## Methods

### Soybean nodulation conditions

Seeds of soybean cultivar ‘Maverick’ and OASS overexpressing transgenic lines (CS02, CS022, and CS027) were germinated on 1% water agar plates in a 30 °C incubator for 3 days. *Bradyrhizobium japonicum* USDA110 was grown in liquid yeast extract-mannitol (YEM). After 4-days growth, the rhizobia were harvested by centrifugation 8,000 × *g* for 15 min. The resulting bacterial pellet was suspended in liquid YEM to a final concentration of 1 × 10^6^ cells/mL. Roots of 3-day-old seedlings were inoculated with *B. japonicum* USDA110 and transferred to autoclaved Leonard jars containing vermiculite. Plants were placed in a growth chamber, which was maintained at a constant temperature of 28 °C with a light intensity of 500 µmol/m^2^/s with a 14 h light period. Nodules were harvested at 15, 20 and 30 days after inoculation and immediately processed for anatomical studies or frozen in liquid nitrogen for biochemical analysis.

### OASS activity assays

Protein extracts from wild-type and transgenic 20 day old nodules were used for measuring OASS activity and its accumulation. Samples from four biological replicates of each sample were pooled together and the assay repeated in triplicate. OASS activity was determined as previously described^31^.

### One and two-dimensional gel electrophoretic separation of soybean nodule proteins

Extraction of soybean nodule proteins and separation by 1- and 2-dimensional SDS-PAGE was performed as described earlier^46^. Stained gels were scanned with an Epson Perfection V700 PHOTO scanner (positive film setting, 24-bit color and scanned at 600 dpi, processed and cropped with PowerPoint.

### Immunoblots

Western blot analysis was performed as described previously^30^. Polyclonal antibodies generated against soybean cytosolic OASS were used for detecting the relative concentration of OASS. Proteins transferred (~20 µg per lane) to the nitrocellulose membranes were incubated overnight with the OASS antibody that was diluted 1:10,000 in Tris-buffered saline (TBS; 10 mM Tris-HCl, pH7.5, 500 mM NaCl) containing 3% (w/v) nonfat powdered milk. Following several washes in TBS-T (TBS containing 0.1% Tween 20), the nitrocellulose membranes were incubated with goat anti-rabbit IgG-horseradish peroxidase conjugate for 1 h. Immunoreactive polypeptides were detected with an enhanced chemi-luminescent substrate (Super Signal West Pico) following the procedure provided by the manufacturer (Pierce Biotechnology, Rockford, IL) and a 1:20,000 ratio. Autoradiographs were scanned with an Epson Perfection V700 PHOTO, with image acquisition done through Adobe Photoshop, and scanned at 300 dpi using 24-bit color picture setting. Images were processed and cropped with PowerPoint.

### Light and transmission electron microscopy

Soybean nodules harvested at 15 and 30 days after inoculation were subjected to light and electron microscopy. The procedure for the embedment of soybean nodules for light and transmission electron microscopy has been described previously^46,47^.

### Analysis of thiol compounds

Cysteine, γ-glutamylcysteine (γGC), glutathione (GSH), and homoglutathione (hGSH) were extracted from plant cell lysates, derivatized with monobromobimane, and were separated by HPLC with quantification as previously described^48^.

### APX, CAT, and SOD activity assays

About 100 mg of 15 and 30 days after inoculation nodules were separately ground to a fine powder under liquid nitrogen with the help of mortar and pestle. To this 1 mL of 50 mM sodium phosphate buffer, pH 7.0 containing 17% (w/v) sucrose was added and thoroughly mixed by vortexing. The slurry was clarified at 400 × g for 10 min at 4 °C and the resulting supernatant was further clarified at 8,000 × g for 20 min at 4 °C in order to remove the bacteroids. Protein concentration in the resulting supernatant, which contains the nodule cytosol, was measured by the method of Bradford (1976). Ascorbate peroxidase (APX) activity assay (total volume 1 mL) contained 50 mM sodium phosphate buffer, pH 7.0, and known amount of nodule cytosol (time = 0) followed by the addition of 0.1 mM H_2_O_2_ and 0.5 mM sodium ascorbate to initiate the assay. The rate of ascorbate oxidation was monitored by a decrease in in optical density (A_290 nm_) over time. An extinction coefficient of 2.8 mM^−1^ cm^−1^ for ascorbate was used to calculate APX activity. Catalase (CAT) activity was based on the rate of decomposition of H_2_O_2,_ which was monitored over time at A_240nm_. The CAT assay was performed in a total volume of 1 mL that contained 50 mM sodium phosphate buffer, pH 7.0, and known amount of nodule cytosol (time = 0), followed by the addition of 0.1% H_2_O_2_ to initiate the reaction. An extinction coefficient of 0.04 mM^−1^ cm^−1^ for H_2_O_2_ was used to calculate CAT activity. Superoxide dismutase (SOD) activity, which was based on the inhibition of nitro-blue tetrazolium (NBT) reduction, was performed as previously described^46^.

### Extraction of metabolites and analysis on LC-MS and GC-MS platforms

Extraction and metabolite analysis were performed essentially as described in previous publications^49,50^. Nodules from individual plants were harvested, freeze-dried and ground to a fine powder using a mortar and pestle with liquid nitrogen. A total of 8 samples (4 biological replications each for transgenic and control plants) were sent to Metabolon Inc., Research Triangle Park, North Carolina for metabolite analysis. A subsample of 20 mg of each sample was thawed on ice and extracted using an automated MicroLab STAR system (Hamilton Company) in 400 mL of methanol containing recovery standards, and then split to three aliquots for analysis by three methods: (1) UPLC-MS performed using a Waters Acquity UPLC (Waters Corporation) coupled to an LTQ mass spectrometer (Thermo Fisher Scientific, Inc.) equipped with an electrospray ionization source; (2) LC methods, chromatographic separation and all scan mass spectra was carried out to record retention time, molecular weight (m/z), and MS/MS^2^ of detectable ions presented in the samples^49^; and (3) GC-MS, bis-trimethyl-silyl-triflouroacetamide-derivatized samples were analyzed on a Thermo-Finnigan Trace DSQ fast-scanning single-quadrupole MS operated at unit mass resolving power^50^. Instrument variability was determined by calculating the median relative standard deviation (RSD) for the internal standards that were added to each sample prior to injection into the mass spectrometers.

### Data imputation, statistical analysis and principle component analysis

Missing values for metabolites were imputed with the observed minimum detected value, based upon the assumption that results were below the instrument limit of detection. Statistical analyses were performed using JMP Version 11 software (SAS Institute Inc., Cary, NC, USA) and “R” (http://cran.r-project.org/), essentially as previously described^49,50^. Principle component analysis, ANOVA and t-tests were performed in JMP software using the normalized data obtained from the LC-MS and GC-MS platforms. For most of the experiments, ten to fifteen nodules were pooled from each of three biological replicates. For metabolite analysis 4 biological replicates were used. All statistical analyses were performed using a confidence threshold of α = 0.05.

## Electronic supplementary material

Supplementary Information

Dataset 1
