# Supplementary material for: Impact of overexpression of cytosolic isoform of O-acetylserine sulfhydrylase on soybean nodulation and nodule metabolome
Source: Sci Rep. 2018 Feb 5;8:2367. doi: 10.1038/s41598-018-20919-8 (PMC5799319; doi:10.1038/s41598-018-20919-8)
Supplement: Supplementary file 1 — Supplementary Information [file 41598_2018_20919_MOESM1_ESM.pdf]

Supplementary Information

Impact of overexpression of cytosolic isoform of *O*-acetylserine sulfhydrylase on soybean nodulation and nodule metabolome

Hari B. Krishnan[1,2]*, Bo Song[1,3], Nathan W. Oehrle[1], Jeffrey C. Cameron[4], Joseph M. Jez[5]

[1]USDA-ARS, Plant Genetics Research Unit, 105 Curtis Hall, University of Missouri, Columbia, MO 65211, USA

[2]Division of Plant Sciences, University of Missouri, Columbia, MO 65211, USA

[3]Key Laboratory of Soybean Biology at the Chinese Ministry of Education, Northeast Agricultural University, Harbin 150030, China

[4]Department of Chemistry and Biochemistry, University of Colorado, Boulder, CO 80309-0596

[5]Department of Biology, Washington University in St. Louis, St. Louis, Missouri 63130, USA

*Corresponding author's E-mail: Hari.Krishnan@ARS.USDA.GOV

Supplemental Fig. 1. Carbon-Nitrogen-Sulfur Balance in soybean nodules. Metabolites that are elevated in OASS overexpressing transgenic soybean nodules are shown in red and those that are reduced when compared to non-transformed wild-type nodules are shown in green fonts.

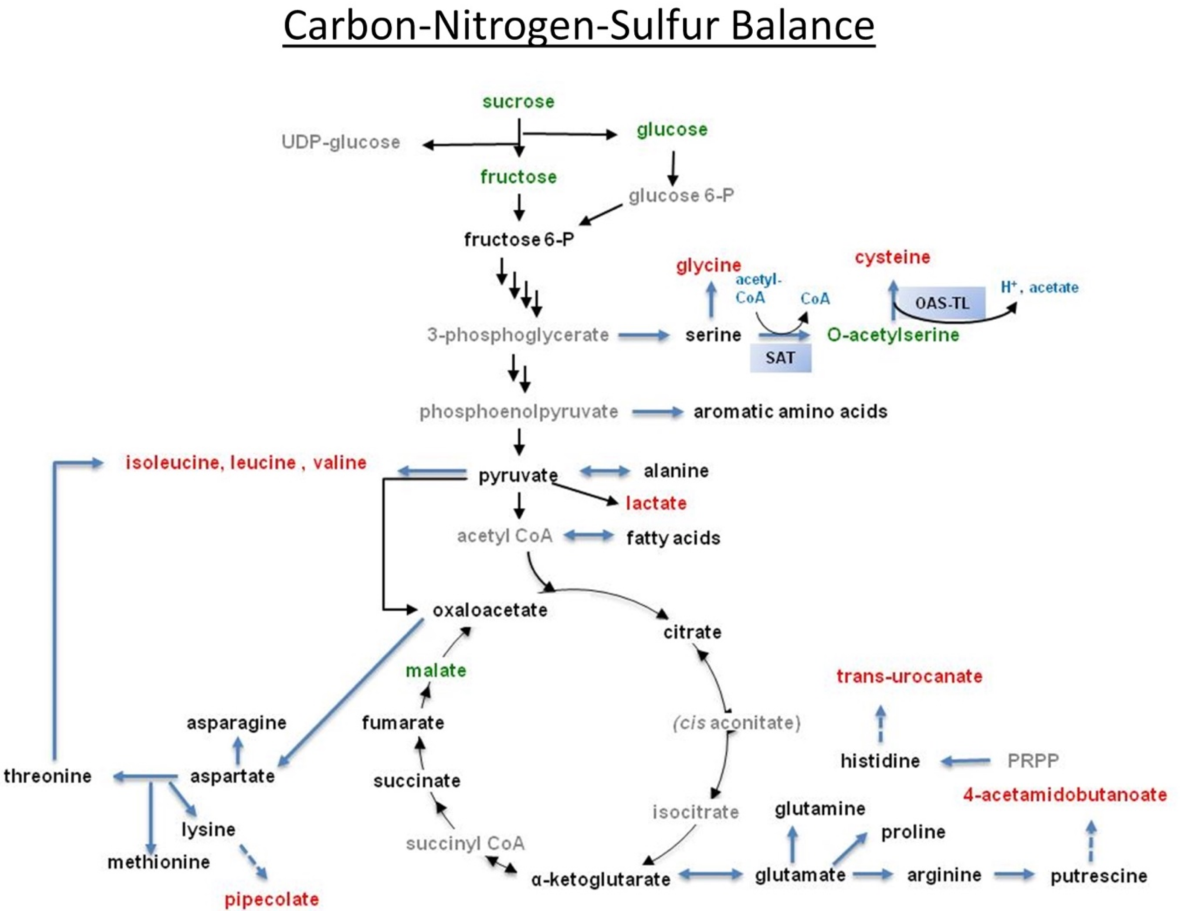

Supplemental Fig. 2. Changes in the abundance of key metabolites involved in sulfur assimilatory pathway in non-transformed wild-type and transgenic soybean nodules. Relative abundance of metabolites is shown on y-axis. Box plots were generated for compounds with significant increase or decrease using both t-test and FDR, with p<0.05 and q<0.10 as significant values.

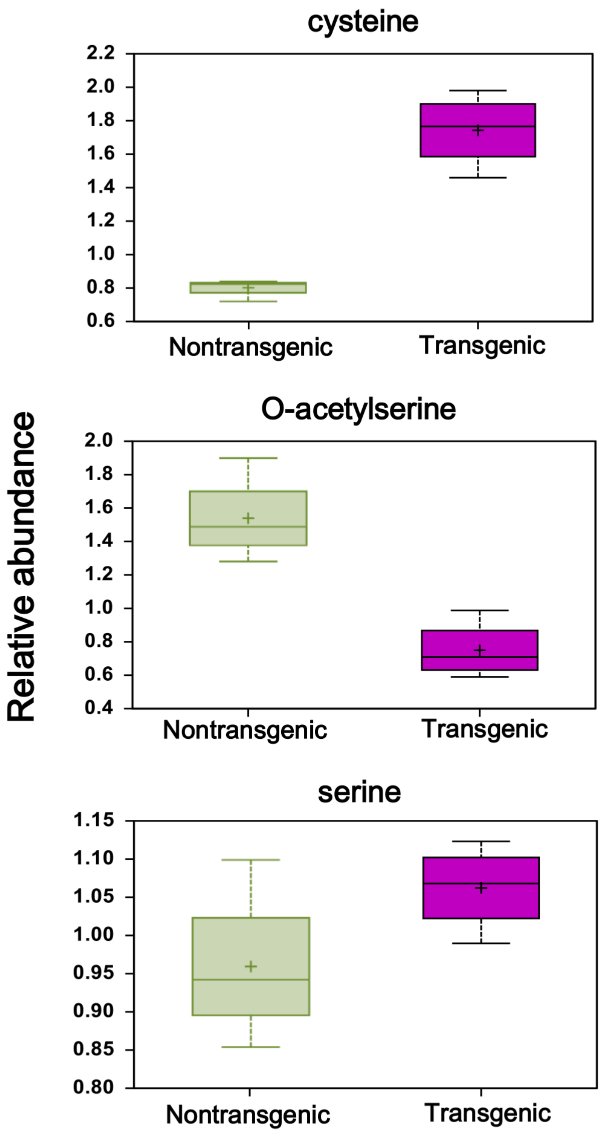

Supplemental Fig. 3. Uncropped digital images of full-length gel and immunoblot. Total nodule proteins from 20 day old nodules of nontransgenic and transgenic plants were separated on 15% SDS-PAGE gels and either stained with Coomassie Blue (A) or transferred to nitrocellulose membrane and probed with antiserum against soybean OASS (B and C). Antibody reactivity was detected by using anti-rabbit IgG-horseradish peroxidase conjugate followed by chemiluminescent detection. Lanes (1 and 5) nodule protein from non-transformed wild-type; lanes (2 and 6), (3 and 7) and (4 and 8) nodule protein from OASS transformed transgenic plants CS02, CS022, and CS027, respectively. Lanes 1 to 4 contain 10 μg and lanes 5to 8 contain 20 μg of nodule protein. Autoradiograph B and C was exposed for 5 and 10 seconds, respectively.

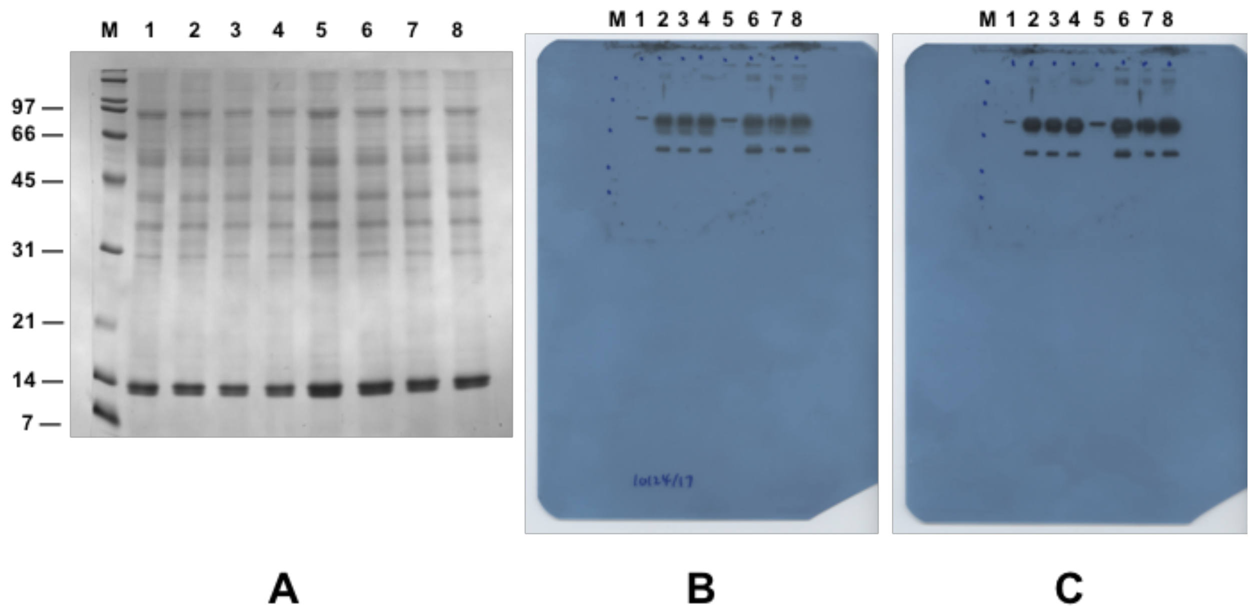

Supplemental Table 2. Antioxidant enzyme activities in 15 and 30 day old soybean nodules*.

	Ascorbate peroxidase	Catalase	Superoxide dismutase
Nontransgenic 15 day	2.41 µmol min^{-1} mg^{-1} ± 0.06	128 µmol min^{-1} mg^{-1} ± 10.39	42.33 U mg^{-1} ± 0.87
Transgenic 15 day	2.31 µmol min^{-1} mg^{-1} ± 0.04	141 µmol min^{-1} mg^{-1} ± 8.78	45.67 U mg^{-1} ± 1.03
	F stat: 2.0773<br>p-value: 0.1688<br>Tukey HSD: 0.1688	F stat: 1.0090<br>p-value: 0.3301<br>Tukey HSD: 0.3301	F stat: 6.0964<br>p-value: 0.0252<br>Tukey HSD: 0.0252
Nontransgenic 30 day	2.27 µmol min^{-1} mg^{-1} ± 0.07	82 µmol min^{-1} mg^{-1} ± 2.89	51.33 U mg^{-1} ± 0.68
Transgenic 30 day	2.44 µmol min^{-1} mg^{-1} ± 0.05	75 µmol min^{-1} mg^{-1} ± 5.86	62.00 U mg^{-1} ± 1.41
	F stat: 4.2503<br>p-value: 0.0570<br>Tukey HSD: 0.0571	F stat: 1.2590<br>p-value: 0.2784<br>Tukey HSD: 0.2784	F stat: 46.6020<br>p-value: 0.0000004<br>Tukey HSD: 0.0010

*Enzyme activity error is reported as standard deviation of the mean. One-way ANOVA with post-hoc Tukey HSD test was performed (n=9). p-values > 0.05 demonstrate no significant difference among enzyme activities.

Supplemental Table 1. Identification of nodule cytosolic proteins. Following 2D-PAGE separation, protein spots were identified using peptide mass fingerprinting (MALDI-TOF-TOF). Spot identifiers correspond to those proteins labeled in Figure 6. MOWSE scores represent those searches performed via Mascot. Peptides matched, % coverage and accession numbers resulted from searches confined to plant databases. Database used was National Center for Biotechnological Information (NCBIprot).

Spot	Protein ID	MOWSE	Peptides Matched (Sequences)<br>----<br>% Coverage	NCBIprot	(Position) Peptide Matched	Peptide Score
**A**	Cysteine synthase (*Glycine max*)	681	7 (6)<br>----<br>32 %	NP_001235628.1	(71-92) K.SVLIEPTSGNTGIGLAFMAAAR.G<br>(71-92) K.SVLIEPTSGNTGIGLAFMAAAR.G + Ox.(M)<br>(176-194) K.IDAFVSGIGTGGTITGAGK.Y<br>(205-225) K.LIGVEPVESPVLSGGKPGPHK.I<br>(265-285) K.EGLFVGISSGAAAAAAFQIAK.R<br>(293-304) K.LIVAVFPSFGER.Y<br>(305-315) R.YLSSVLFESVR.R	100<br>98<br>121<br>170<br>168<br>55<br>67
**B**	Phosphinothricin acetyl transferase (synthetic construct)	275	4 (4)<br>----<br>32 %	AAR86773.1	(38-52) R.TEPQEPQEWTDDLVR.L<br>(81-96) R.NAYDWTAESTVYVSPR.H<br>(100-112) R.TGLGSTLYTHLLK.S<br>(121-135) K.SVVAVIGLPNDPSVR.M	57<br>63<br>74<br>82
